# Laparoscopic Pyloromyotomy: A Modified Simple Technique

**Published:** 2016-01-01

**Authors:** Mohammed Omer Anwar, Yasser Al Omran, Saeed Al-Hindi

**Affiliations:** 1 Barts and the London School of Medicine and Dentistry, Garrod Building, Turner Street, Whitechapel, London, E1 2AD, United Kingdom; 2 Salmaniya Medical Complex, Manama, Kingdom of Bahrain

**Keywords:** Infantile hypertrophic pyloric stenosis, Laparoscopy, Ophthalmic knife, Pyloromyotomy

## Abstract

Background: A modified laparoscopic pyloromyotomy (LP) technique may provide an alternative to treating infantile hypertrophic pyloric stenosis (IHPS) by improving operative timing with reduction of postoperative complication rates, compared with a three-port trocar system.

Methods: Thirty-three infants were treated with IHPS at a single-centre between January 2002 and December 2011. The local surgical incision to the pylorus was performed according to Ramstedt’s pyloromyotomy; but with a two-port trocar system (umbilical and right lower abdominal crease ports), following a controlled stab wound into the epigastric region and a 3mm incision to allow introduction of ophthalmic knife. With the aid of atraumatic forceps and camera guidance, the ophthalmic knife was used to carefully incise the seromuscular layer, which allows improved manual tactile sensation compared to ergonomic laparoscopic spreaders. A Benson pyloric spreader was then used to further separate the pyloric muscle layer to complete the procedure.

Results: In all 33 infants treated, LP was safely performed with no evidence of duodenal or mucosal perforation with complete pyloromyotomy achieved in each case. The postoperative course was rather uneventful apart from an umbilical wound infection.

Conclusion: This modified approach is simple, safe and allows improved operative timing, whilst increasing surgeon’s confidence by tactile sensation.

## INTRODUCTION

The surgical treatment to relieve infantile hypertrophic pyloric stenosis (IHPS) was famously introduced by Conrad Ramstedt in 1911. Ramstedt’s pyloromyotomy consisted of an open right upper quadrant (RUQ) incision towards the supra-umbilical approach [1]. Since then, Tan and Bianchi [2] in 1986 proposed a new open semi-circumumbilical (UMB) incision for improved cosmesis, followed by Alain et al [3] who introduced the first laparoscopic procedure report in 1991.

Since 2002, we have performed LP, which is consistent with Ramstedt’s technique, through a non-conventional two-port trocar system. In replacement of the third trocar, a controlled epigastric stab wound of a 3mm size allowed instrumental access to the hypertrophied pyloric muscle. Through this wound, an ophthalmic knife and Benson pyloric spreader were used to complete the pyloromyotomy. This report discusses a new simple technique combining laparoscopy with surgical instruments, commonly required in open procedures to allow completion of pyloromyotomy and its results in 33 infants.

## MATERIALS AND METHODS

Thirty-three infants treated for IHPS via a simple modified technique for pyloromyotomy by a single paediatric surgeon at a single centre were included in the study. Three other patients, two infants with metabolic disease and one infant with cardiac disease were excluded. Most of the patients (82%) were males. The mean weight was 4.8 kg (range 3-5.2 kg) and the mean was 45 days age varied from (range 3-55 days). The diagnosis of IHPS was made upon clinical history, examination and confirmed through ultrasound scan. For every case, consent was gained from the patient’s parents or guardians, once the benefits and potential complications of the procedure were discussed. All infants were kept nil by mouth (NBM) for 8 hours pre-operatively with IV fluid access and tests were ordered such as complete blood count and urea and electrolytes.

All procedures were performed under general anaesthesia with the patient lying in the supine position. Our surgical technique was standardised for each case, with regards to trocar size, trocar site position, same surgical instruments and single paediatric surgeon. 

**Surgical Procedure**

After draping, a 5mm incision was created to introduce a trocar through an infra-umbilical skinfold. Pneumoperitoneum was produced by the classic open Hasson technique. Insufflation of CO2 was started at 0.1 litre/min and was increased to 1 litre/min, with the target pressure kept between 8 and 10mmHg. A 30 degree laparoscopic camera was then placed through the infra-umbilical skinfold. A second trocar was introduced under camera vision in the right lower abdominal skin crease by 5mm incision to allow insertion of forceps for atraumatic grasping of the duodenum; this was to establish stabilisation of the pylorus (Fig. 1). A 3mm local stab wound was formed in the epigastric area, 1cm left of the midline, with an ophthalmic surgical knife, which penetrated the full thickness of the skin under camera guidance (Fig. 2). The size of the blade used was 2.8mm with its length corresponding to 12cm. In order to maintain the pneumoperitoneum, we used a temporary purse-string suture to control leakage of air around the epigastric wound. The ophthalmic knife was used to carefully cut the seromuscular layer of the pylorus. Once the ophthalmic knife was removed, the purse-string suture was pulled together and maintained the pneumoperitoneum to allow subsequent introduction of the Benson pyloric spreader (Fig. 3). The purse-string suture was 5mm around the epigastric stab wound using 4/0 nylon to control air leak during insertion and removal of instruments through the epigastric stab wound. The two ends of the suture will hold the wound site by force. Therefore the wound will be tight by pulling both ends and loose by releasing and pull method. 

**Figure:**
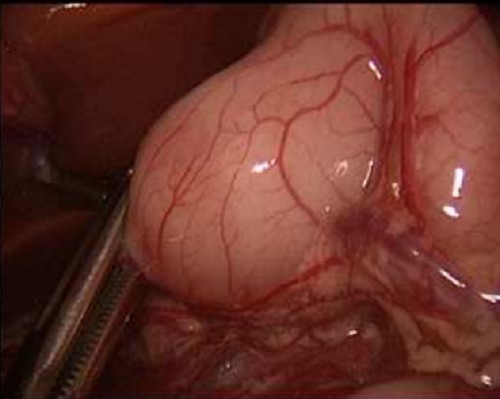
Figure 1: Shows atraumatic grasping of the duodenum using forceps.

**Figure:**
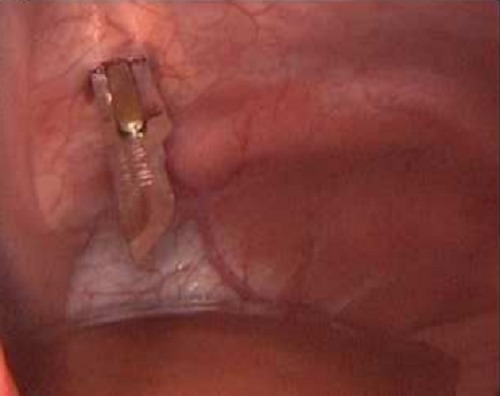
Figure 2: A controlled stab incision of the ophthalmic surgical knife through the abdominal wall.

**Figure:**
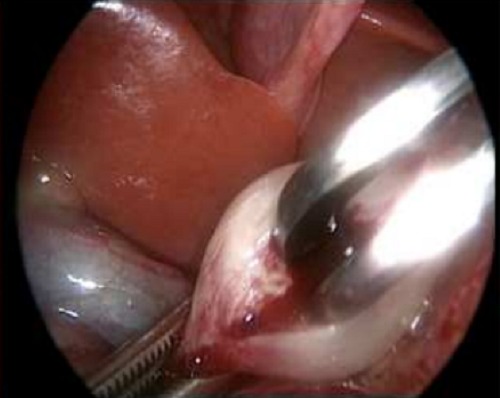
Figure 3: Introduction of the Benson Pyloric Spreader.

The ophthalmic knife provided easier handling to incise the seromuscular layer partially where the Benson pyloric spreader was then used to complete the dissection to the depth of the submucosa. The operation was then continued according to Ramstedt’s pyloromyotomy using same surgical instruments, when performing the traditional open technique. Once the pylorus was stabilised by forceps, the Benson pyloric spreader was used to separate the pyloric muscle, until the mucosa bulged out. After completion of the pyloromyotomy, the spreader was removed back through epigastric wound. The mucosa was assessed for perforation by inflating the stomach with 50cc air via a nasogastric (NG) tube. The abdominal wounds were then closed. No antibiotics were used during the operation and the NG tube was used for gastric decompression for 8hrs postoperatively.

## RESULTS

The operation was completed successfully with minimal episodes of blood loss in all the 33 infants. The patients and their medical records were assessed for the following: postoperative emesis, mucosal perforation, duodenal perforation and wound implications. One patient developed an umbilical wound infection (incidence of 3%). No other adverse outcomes were recorded. The mean operative time was 23 min (range 20-25 min). Feeds were gradually started after 8hrs of the operation, reaching full feeds after 24hrs. The mean length of postoperative stay was 48hrs (range 24-76hrs). At a median 6-month follow-up, all infants were thriving well with barely visible small laparoscopy scars to report on the abdomen. 

## DISCUSSION

Many paediatric surgeons have adopted LP recently, though the classic open procedures still stays the mainstay of treatment in the developing world. Alain et al performed the first laparoscopic technique in 1991 [3]; this was followed by many other reports/ series [4-6]. 

In our series, the surgical approach was modified with introduction of the second trocar port into the right lower abdominal crease, as opposed to the conventional approach through a right upper quadrant incision to supra-umbilical approach [7]. This allowed for an easier approach to the stomach and duodenum, with better postoperative cosmesis at the entry sites for incision and avoided the risk of inadvertent seromuscular lacerations from a right hypochondriac approach, which have been reported in up to 30% of cases [8]. Furthermore, we initiated a controlled stab wound of 3mm into the epigastric region with an ophthalmic knife and Benson pyloric spreader to complete the pyloromyotomy. This provided the benefit of using instruments commonly used in open procedures to allow further control whilst manipulating the hypertrophied pyloric muscle. We found that these instruments provided a feeling of normal tactile sensation and force, as opposed to utilising laparoscopic equipment where flexibility can affect control during incision and spread of the muscle. 

There have been other studies recently in the literature that have demonstrated the role of single incision laparoscopic surgery (SILS) to complete pyloromyotomy [9,10]. Here, a single trocar was inserted through an umbilical incision to deploy working instruments into the abdomen. The reason as to why a single trocar through the umbilicus wasn’t used in our study for the deployment of working instruments; was the increased chance of collision between working instruments particularly with an already limited workspace inside the infant. The main advantage in utilising two trocars at a good distance from each other is that it will reduce the incidence of collision between instruments by providing more space and there will be adequate control to stabilise the pylorus in a shorter operative time. 

Bertozzi et al. [1], performed laparoscopic-assisted pyloromyotomy (LAP) technique with a 12mm Hasson trocar. Once abdominal exploration had been performed, the authors described how the pylorus was exteriorised through a right umbilical incision, where Ramstedt’s pyloromyotomy was performed. Initially, the trocar was removed to allow full extent of delivery of the pylorus through the umbilicus. It was then re-inserted after pylorus was reintroduced into abdomen following pyloromyotomy. This may present with a few problems such as trying to re-establish the correct pneumoperitoneum to avoid technical difficulties, whilst assessing mucosal integrity through insufflation of air via an NG tube. Furthermore, inserting such a large trocar has its own risks of causing damage to abdominal structures potentially leading to abdominal wall haematomas, umbilical wound infections, umbilical hernia or penetration of important mesenteric vessels to the small or large intestine [11]. 

Bufo et al. initially described a safe laparoscopic technique by the use of an arthroscopy knife and specialised laparoscopic spreader [4]. However, we used an ophthalmic knife for better comfort and control to handle careful dissection of the hypertrophied muscle. Although ergonomically designed laparoscopic spreaders are effective to provide a more complete pyloromyotomy with a lowered incidence of mucosal perforations, the Benson pyloric spreader has a markedly improved manual tactile sensation [12]. In our case series, there was no evidence of mucosal tears or injuries as we manipulated the pyloric muscle fibres and this was confirmed by insufflation of 50cc air into the stomach via a NG tube. Overall, Bufo et al. reported a wound complication rate of 4.3%, while we had an umbilical infection of 3%, which subsequently resolved after antibiotics [4]. 

Turial et al. reported a novel advancement towards using micro-laparoscopic pyloromyotomy in a feasibility study [13]. The authors concluded that surgical instruments should be downgraded in the paediatric patient to minimise trauma via instrumental portal access and increase peri- and post-operative patient comfort. The authors were concerned about the incidence of incomplete pyloromyotomy which ranged between 1.4% and 5.6% in previous studies. Therefore, the authors decided to implement micro-cautery to dissect the seromuscular layer in a singular in-depth plane [13]. Whilst their initial results look promising with optimal cosmesis, further studies in the literature would be required to establish rates of mucosal perforation, postoperative emesis or wound complications.

## CONCLUSION

The operative time in our series is slightly improved due to quicker pre-surgical preparation time whilst initiating two trocars as opposed to the conventional three trocar-port system along with minimal minor complication rate; further data would ideally be required with patients in multiple institutions. The only technical difficulty is added efforts to maintain the pneumoperitoneum through the epigastric port, whilst removing the ophthalmic knife and inserting the Benson pyloric spreader. We find this technique simple, easy, and practical. 

## Footnotes

**Source of Support:** None

**Conflict of Interest:** None
