# Laparoscopic Pyloromyotomy: A Modified Simple Technique

**Published:** 2016-04-10

**Authors:** Mohammed Omer Anwar, Yasser Al Omran, Saeed Al-Hindi

**Affiliations:** 1 Barts and the London School of Medicine and Dentistry, Garrod Building, Turner Street, Whitechapel, London, E1 2AD, United Kingdom; 2 Salmaniya Medical Complex, Manama, Kingdom of Bahrain

## Erratum

It is regretted that the first and last names for the author Yasser Al Omran was published wrong for the manuscript entitled "Laparoscopic Pyloromyotomy: A Modified Simple Technique" published as original article in Journal of Neonatal Surgery volume 5 issue 1. The corrected first name is “Yasser” and last name is “Al Omran”. All the authors contributed equally in the manuscript and share equal first authorship where applicable.

